# COPB2: a transport protein with multifaceted roles in cancer development and progression

**DOI:** 10.1007/s12094-021-02630-9

**Published:** 2021-06-08

**Authors:** Y. Feng, X. Lei, L. Zhang, H. Wan, H. Pan, J. Wu, M. Zou, L. Zhu, Y. Mi

**Affiliations:** 1 Wuxi Medical College, Jiangnan University, Wuxi, 214122 Jiangsu Province China; 2 Department of Urology, Affiliated Hospital of Jiangnan University, Wuxi, 214122 Jiangsu Province China; 3 Department of Urology, Affiliated Changzhou No. 2 People’s Hospital of Nanjing Medical University, Changzhou, 213003 Jiangsu Province China; 4 Department of Burns and Plastic Surgery, Affiliated Hospital of Jiangnan University, Wuxi, 214122 Jiangsu Province China; 5 Wuxi Clinical Medicine School of Integrated Chinese and Western Medicine, Nanjing University of Chinese Medicine, Wuxi, 214122 Jiangsu Province China

**Keywords:** COPB2, Cancer, Proliferation, Survival, Tumorigenesis, Invasion, Metastasis

## Abstract

The Coatomer protein complex subunit beta 2 (COPB2) is involved in the formation of the COPI coatomer protein complex and is responsible for the transport of vesicles between the Golgi apparatus and the endoplasmic reticulum. It plays an important role in maintaining the integrity of these cellular organelles, as well as in maintaining cell homeostasis. More importantly, COPB2 plays key roles in embryonic development and tumor progression. COPB2 is regarded as a vital oncogene in several cancer types and has been implicated in tumor cell proliferation, survival, invasion, and metastasis. Here, we summarize the current knowledge on the roles of COPB2 in cancer development and progression in the context of the hallmarks of cancer.

## Introduction

Cancer remains a huge global health problem. Based on data from the International Agency for Research on Cancer, 1,898,160 new cancer cases and 608,570 cancer deaths were reported worldwide in 2021 [1], and the global cancer burden is expected to reach 28.4 million cases by 2040 [2]. The situation in China is particularly severe, with both the number of new cases and deaths ranking first in the world, which highlights the need to develop therapy for all types of cancer [3].

Due to the variety of cancer research, we decide to discuss from a different perspective at the cellular level. In eukaryotic cells, a large number of proteins and lipids are transported through transport vesicles to various organelles and the cell surface, so they can perform their physiological functions. Despite their pathogenic properties, cancer cells have the same intracellular machinery as normal cells, at least for a certain period of time, which suggests the importance of coat proteins (COPs) in cancer cells, as well as in normal cells. COPs play an important role in vesicular transport, and they can be classified into three types: clathrin, COPI, and COPII [4]. COPI consists of seven subunits: α-COP, β-COP, β’-COP, γ-COP, δ-COP, ε-COP, and z-COP. It carries cargo molecules, such as proteins and lipids, from the Golgi to the endoplasmic reticulum (ER) and mediates the reverse and forward transport of materials between the Golgi membrane and vesicles, thereby maintaining the polarity of the Golgi structure and the maturity of membrane vesicles [5–8].

Of the seven subunits that form COPI, COPB2 (also known as COPI coat complex subunit beta 2, β’-COP, P102 or coatomer protein complex subunit beta prime) [6], in particular, has been shown to have a high correlation with tumors. *COPB2*, which is located on chromosome 3q23, encodes a protein with 906 amino acids (102.5 kDa) [9, 10]. It is mainly distributed in the ER, the Golgi stack membrane, and COPI membrane vesicles, and it is involved in intracellular protein transport, ER-to-Golgi vesicle-mediated transport, regressed membrane vesicle-mediated transport, Golgi-to-ER transport, inner Golgi-to-ER vesicle-mediated transport, and so on [6, 7, 11].

As a coatomer protein, COPB2 plays a major role in embryonic development and tumor progression and is associated with multiple pathological processes. Current studies have demonstrated that *COPB2* is a vital oncogene in many cancer types due to its ability to regulate the proliferation, survival, tumorigenesis, invasion, and metastasis of cancer cells. In this paper, we focus on the emerging roles of *COPB2* in cancer development and progression in the context of the hallmarks of cancer. Through this comprehensive review, we discuss the accumulating evidence for the future clinical utilization of *COPB2* as a therapeutic target and a biomarker. We also provide insights that can open new avenues for studying the role of *COPB2* in cancer.

## Functions associated with COPB2

**Fig. 1:**
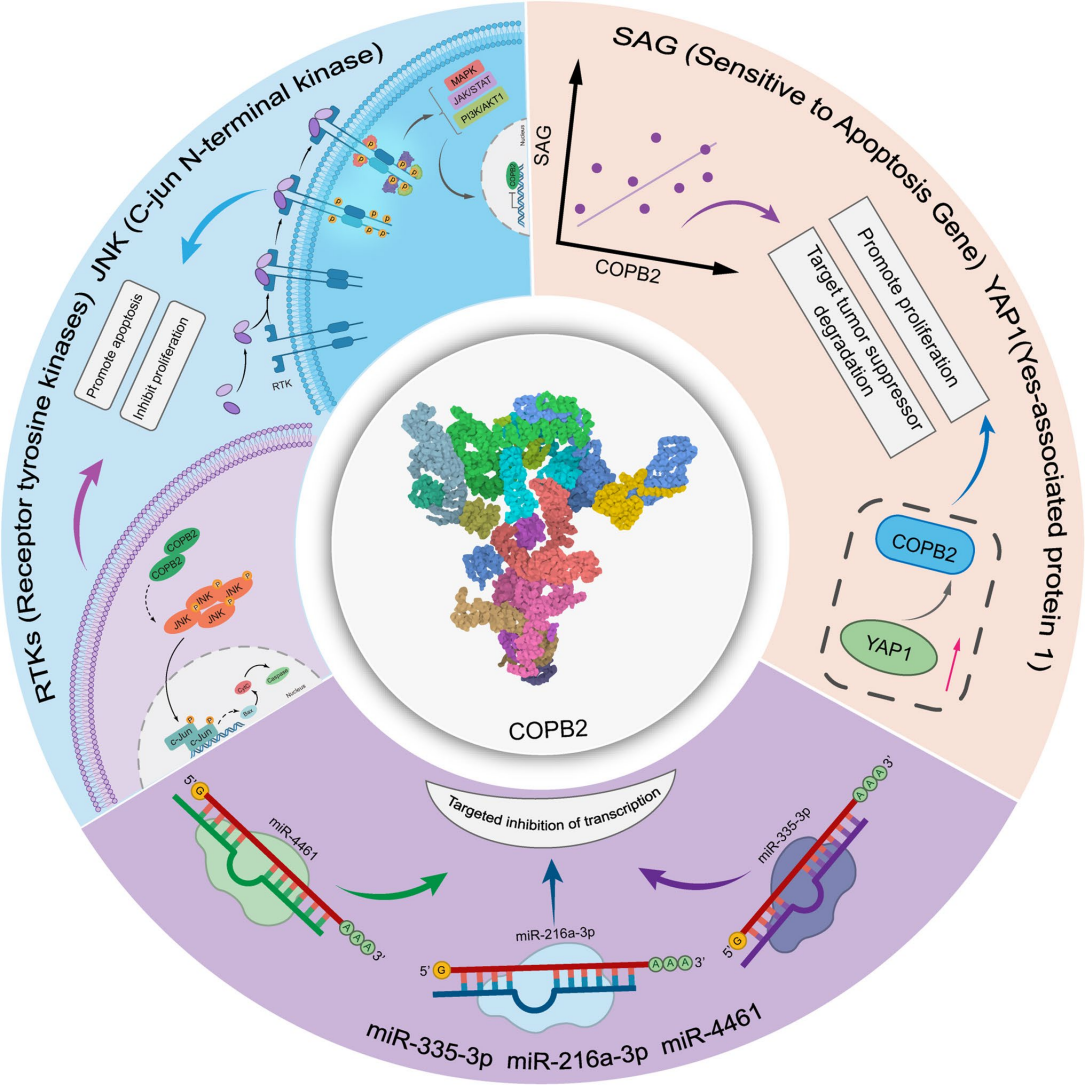
The mechanism functions associated with COPB2. There is a positive correlation between SAG and COPB2 expression, the downregulation of SAG or COPB2 and upregulating YAP1 expression promoted cancer cell proliferation and tumorigenesis; COPB2 promote tumor cell apoptosis and inhibit tumor formation through activating the RTKs signaling pathways and JNK/c-Jun signaling pathways after silencing; miR-335-3p, miR-216a-3p and miR-4461 inhibit the function of COPB2 by targeting 3′UTR of COPB2

Several studies have reported direct and indirect associations between *COPB2* and cancer. *COPB2* overexpression has been reported in various kinds of cancers (Table 1). Generally, the involvement of *COPB2* in tumor progression has been found to be related to the regulation of upstream genes, such as the *Sensitive to apoptosis gene* (*SAG* or *RNF7*) [12] and *Yes-associated protein 1* (*YAP1*) [13]; the activation of receptor tyrosine kinase (RTK) [14] and c-jun N-terminal kinase (JNK)/c-Jun signaling pathways [15]; and the targeting of microRNAs [16–18] (Fig. 1).

**Table 1:** The cancer types and cell function experiments associated with COPB2

The articles	Cancer type	Cell lines	Cell function					
			Proliferation	Apoptosis	Invasion and metastasis	Tumorigenesis	Cell cycle	The expression of COPB2 and others
Bhandari et al. [36]	Breast cancer	MDA-MB-231*, BT-549*, SK-BR-3, BT-474, MCF-7, MCF-10A	CCK-8	si-RNA Apoptosis Assay	Invasion and Metastasis Assay	NA	NA	WB
Pu et al. [16]	Lung Adenocarcinoma Cancer	Lung adenocarcinoma cell lines: H1299, A549, SK-MES-1, H1688, H1975*	MTT	FCM	NA	NA	NA	WB; qRT-PCR
Li et al. [35]	Cholangiocellular Carcinoma	RBF* QBC939*	NA	Annexin V-APC Apoptosis Assay	NA	NA	FCM	WB
Mi et al. [34]	Prostate Cancer	PC-3*, DU-145, CWR22RV1, LNCaP	GFP-based Imaging; Colony Formation Assay	FCM	NA	NA	FCM	WB; qRT-PCR
An et al. [17]	Gastric Cancer	Normal gastric mucous membrane epithelial cell line: GES‑1; Gastric cancer cell lines, BGC‑823*, SGC‑7901, MGC‑803, MKN45	MTT; BrdU incorporation; Colon Formation Assay	FCM	NA	Tumorigenesis in nude mice and in vivo imaging	NA	qRT-PCR
Mi et al. [44]	Prostate Cancer	Prostate Carcinoma Cell Line: CWR22RV1*	CCK-8; Colon Formation Assay	FCM	NA	NA	FCM	WB; qRT-PCR
Wang et al. [4]	Colon Cancer	Six human CRC cancer cell lines: RKO*, SW480, HCT116*, DLD1, HT‑29, SW620	MTT; Colon Formation Assay	NA	NA	NA	FCM	WB; qRT-PCR
Chen et al. [19]	Colorectal Cancer	Human normal colorectal mucosal cell: FHC; Colorectal Cancer Cell Lines: DLD1*, HCT116*, SW480*	CCK-8	NA	Transwell Assay	NA		WB; qRT-PCR; Dual-luciferase Reptorer Assay
Pu et al. [21]	Lung Adenocarcinoma cancer	Human bronchial epithelial cells: BEAS-2B (CRL-9609); Human lung adenocarcinoma cell lines NCI-H1299 (CRL-5803, A549 (CCL-185), SK-MES-1 (HTB-58), NCI-H1688 (CCL-257), NCI-H1975 (CRL-5908)*	CCK-8	FCM	Transwell Assay	NA	FCM	WB; qRT-PCR
Liu et al. [15]	Breast Cancer	The normal breast cell line: MCF-10A; The breast cancer cell lines: MCF-1, SK-BR-3*, T-47D	CCK-8	Migrated Cells Following Transfection	NA	NA	NA	qRT-PCR
Wang et al. [20]	Lung Cancer	Normal human bronchial epithelial cell line HBE:ml055209 Human LUAD Cell Lines: NCI-H1299 (CRL-5803), A549 (CCL-185)*, and H1975 (CRL-5908)*	MTT; Colon Formation Assay	NA	Transwell Assay	NA	FCM	WB; qRT-PCR

*WB* Western Blot Assay, *FCM* Flow Cytometry Assay, *qRT-PCR* Real-time Quantitative PCR

*Used for follow-up studies of selected cancer cells lines; Others: The expression of the up-regulation or down-regulation of COPB2; NA: Not available

SAG is an oncoprotein that targets several tumor suppressors for degradation [19–21] and is positively correlated with COPB2 expression, which suggests the potential oncogenic effects of *COPB2* [12]. Similarly, Pu et al. [13] reported that COPB2 can promote the proliferation of lung cancer cells by upregulating the expression of YAP1, another oncoprotein that contributes to tumorigenesis as a downstream effector in the tumor-suppressive Hippo pathway [22, 23].

Because *COPB2* is overexpressed in several types of malignant tumors, COPB2 knockdown or silencing would help determine its role in cancer. An et al. [14] tried to determine the significance and function of COPB2 in gastric cancer using a *COPB2* knockdown model, which revealed an association with the RTK signaling pathway and downstream signaling cascade molecules. RTKs are type I transmembrane proteins that can modulate fundamental cellular functions, including cell division, growth, metabolism, differentiation, migration, and survival by activating a wide range of downstream signaling cascades [24]. RTKs participation has also been reported in the development and progression of human cancer via gain-of-function mutations, genomic amplification, chromosomal rearrangements, and autocrine activation [25]. The knockdown of COPB2 in gastric cancer cell lines suppressed colony formation and promoted apoptosis via the inhibition of RTK signaling and downstream signaling cascade molecules, which suggests that COPB2 is a potential target for gene silencing for the treatment of gastric cancer [14]. The JNK/c-Jun signaling pathway was also activated by COPB2 silencing in colorectal cancer (CRC) [15]. JNK proteins are a subgroup of MAPK with conservative evolution in higher animals. They promote tumor cell apoptosis and inhibit tumor formation by promoting the transcription of apoptotic target genes and the expression of apoptotic proteins [26, 27]. Thus, COPB2 silencing inhibited CRC cell proliferation and induced apoptosis via the JNK/c-Jun signaling pathway.

As knowledge regarding the functions of exosomes grew, the understanding of their roles in cancer has likewise deepened. When investigating the function of bone marrow-derived mesenchymal stem cell (BMSC)-derived exosome miR-4461 in CRC, Chen et al. [16] found that *COPB2* mRNA levels negatively correlated with the levels of miR-4461. Further studies revealed that the BMSC-derived exosome miR-4461 downregulated COPB2 and inhibited cell migration and invasion. Similar to the observations on miR-4461 in CRC, miR-335-3p and miR-216a-3p have been found to target the 3′UTR of *COPB2*, which led to the inhibition of COPB2 in lung adenocarcinoma (LUAD) [18] and lung cancer [17] cell lines, respectively.

### COPB2 and cancer cells

Cancer is caused by genetic mutations in cancer cells [28]. Cancer progression is highly complex and is characterized by several hallmarks, including uncontrolled proliferation, insensitivity to growth-inhibitory (antigrowth) signals, evasion of apoptosis, limitless replicative potential, sustained angiogenesis, tissue invasion, and metastasis [29]. *COPB2* involvement has been reported as an oncogene in some of these mechanisms, especially in proliferation, apoptosis, invasion, migration, cell cycle, and tumorigenesis. In the following sections, we describe the roles of *COPB2* in each of these processes.

### COPB2 and the proliferation of cancer cells

Telomeres become shorter with each round of cell division (mitosis) [30]. When the telomeres have been reduced to a certain length, cells can no longer maintain chromosomal stability and cellular activity, and they eventually die [31]. With the activation of telomerase, the length of the telomere is maintained, which promotes the immortalization of cells. Subsequently, the cells gain the ability to proliferate without limit and transform into cancer cells.

Two main types of genes regulate cell growth. Proto-oncogenes are involved in promoting cell growth and mitosis, whereas tumor-suppressive genes are responsible for inhibiting cell growth or regulating cell division. *COPB2* is involved in tumorigenic processes as a proto-oncogene that has been implicated in the proliferation of cancer cells. Mi et al. [32] first demonstrated the effect of COPB2 on the proliferative ability of prostate cancer cell lines by showing that the downregulation of COPB2 inhibited cell proliferation. The research of Wang et al., Li et al., and Bhandari et al. [4, 33, 34] also showed similar involvement of *COPB2* in colon cancer, cholangiocellular carcinoma, and breast cancer.

Other studies have indicated that COPB2 is involved in the proliferation of cancer cells by disrupting relevant signaling pathways. For instance, An et al. [14] showed that COPB2 was involved in the pro-proliferative effects of the RTK signaling pathway in gastric cancer. Similarly, Liu et al. [12] demonstrated that COPB2-related signaling was involved in the pro-proliferative effects of *SAG* in breast cancer.

The first human disease known to be associated with miRNA dysregulation was chronic lymphoblastic leukemia; a number of other miRNAs have since been associated with cancer [35, 36]. Chen et al. and Wang et al. [16, 17] demonstrated that the proliferation of CRC cells results from the interaction between miR-4461/miR-216a-3p and the proto-oncogene *COPB2*. In addition, the effects of COPB2 and miR-335-3p were observed in lung cancer, where miR-335-3p mimics significantly increased the proliferation of lung cancer cells following COPB2 knockdown [18]. Because malignant proliferation of cancer cells is the most important mechanism underlying tumor formation, controlling the proliferation of cancer cells by regulating *COPB2* would be a major step in the treatment of cancer.

### COPB2 and cancer cell apoptosis

There are two main types of cell death: necrosis and apoptosis. The main goal of traditional tumor therapy is to use cytotoxic drugs or radiation to cause necrosis. Apoptosis, or programmed death, is a gene-mediated process of suicide. Not only is it the opposite of cell proliferation and mitosis as in terms of function, but it is also a mechanism for removing excessively damaged and precancerous cells. Genes that have been associated with apoptosis include *TP53* (encodes p53) [37], *MYC* (encodes c-Myc) [38], *BCL2* (encodes B-cell lymphoma 2, Bcl-2) [39], *COPB2*, and others [32, 40–43]. Silencing COPB2 greatly affects the apoptotic ability of cancer cells. Mi et al. [44] suggested that COPB2-targeted siRNA (siCOBP2) promoted cancer cell apoptosis. Li et al. [33] have also shown that knocking down COPB2 promotes apoptosis in human RBE cholangiocellular carcinoma cells. Similarly, Wang et al.’s [15] study showed that knocking down COPB2 promoted apoptosis in human colon cancer cells.

COPB2 silencing also promotes the activation of the RTK [14] and JNK/c-Jun [15] signaling pathways in gastric cancer and CRC. COPB2 is also involved in cancer cell apoptosis by targeting downstream microRNAs. The rate of apoptosis in LUAD cell lines significantly increased after *COPB2* knockdown via RNA silencing, and miR-335-3p [18] and miR-216a-3p [17] significantly increased the effects of siCOPB2. Understanding the relationship between COPB2 and cancer cell apoptosis provides new strategies for the diagnosis and treatment of cancer and highlights the potential of COPB2 as a new biomarker for the progression of cancer and monitoring treatment effects.

### COPB2 and the invasion and migration of cancer cells

Invasion and migration of cancer cells result from the deterioration of tumor lesions and the accumulation of malignant properties, which are signs of late stages in the progression of malignant tumors [45]. During this process, malignant cells dissociate from the original tumor mass, reorganize their attachment to the tumor extracellular matrix (ECM) though alterations in cell–ECM adhesion dynamics, and start degrading the surrounding ECM to eventually invade through adjacent tissues and/or intravasate into blood vessels and travel through the circulation to distant sites in the body [46]. *COPB2* also plays an important role in controlling the invasive ability of cancer cells. For instance, Bandari et al. [34] showed that downregulating COPB2 significantly inhibited the migratory and invasive capacities of breast cancer cells. Based on the study by Liu et al. [12], knocking down either *SAG* or *COPB2* significantly inhibited breast cancer cell migration and invasion. The migratory and invasive capacities of CRC and lung cancer cells decreased upon treatment with siCOPB2 or with siCOPB2 plus miR-4461 [16] and miR-216a-3p [17] mimics, respectively.

### COPB2 and the cancer cell cycle

The cell cycle is a series of physiological processes that lead to cell division [47]. Cell cycle regulation has two main mechanisms, namely, cell cycle-driven mechanisms and regulatory mechanisms. When the cell cycle regulatory mechanism is disrupted, normal cell growth becomes uncontrollable, and normal cells are transformed into tumor cells. The cell cycle is divided into four consecutive periods: G1, S, G2, and M [48, 49]. The G1 phase of the cell cycle is controlled by an event known as a restriction point; when the restriction point control becomes non-functional for any reason, uncontrolled proliferation occurs in cancerous cells [50]. Regulating gene expression to control the cell cycle is instructive and meaningful for the treatment of tumors. Mi et al. [32] have demonstrated that prostate cancer cell lines were arrested in the G1 phase after *COPB2* knockdown, which, in turn, promoted tumorigenesis. Li et al. [33] found that downregulation of COPB2 arrested the cell cycle in the G1 phase in human cholangiocellular carcinoma cells. Furthermore, in a study by Wang et al. [4], silencing *COPB2* induced G1 phase arrest and inhibited cell cycle progression in RKO CRC cells; in contrast, HCT116 human CRC cells were arrested at the S phase following COPB2 silencing.

### COPB2 and tumorigenesis

*COPB2* has been found to be upregulated in all kinds of cancer tissue. A study has demonstrated that COPB2 promoted tumorigenesis through the downregulation of YAP1 [33]. Additionally, knockdown of *COPB2* significantly downregulated the expression (in varying degrees) of phosphorylated target factors in the RTK signaling pathway [14].

### COPB2 protein interactions

**Fig. 2:**
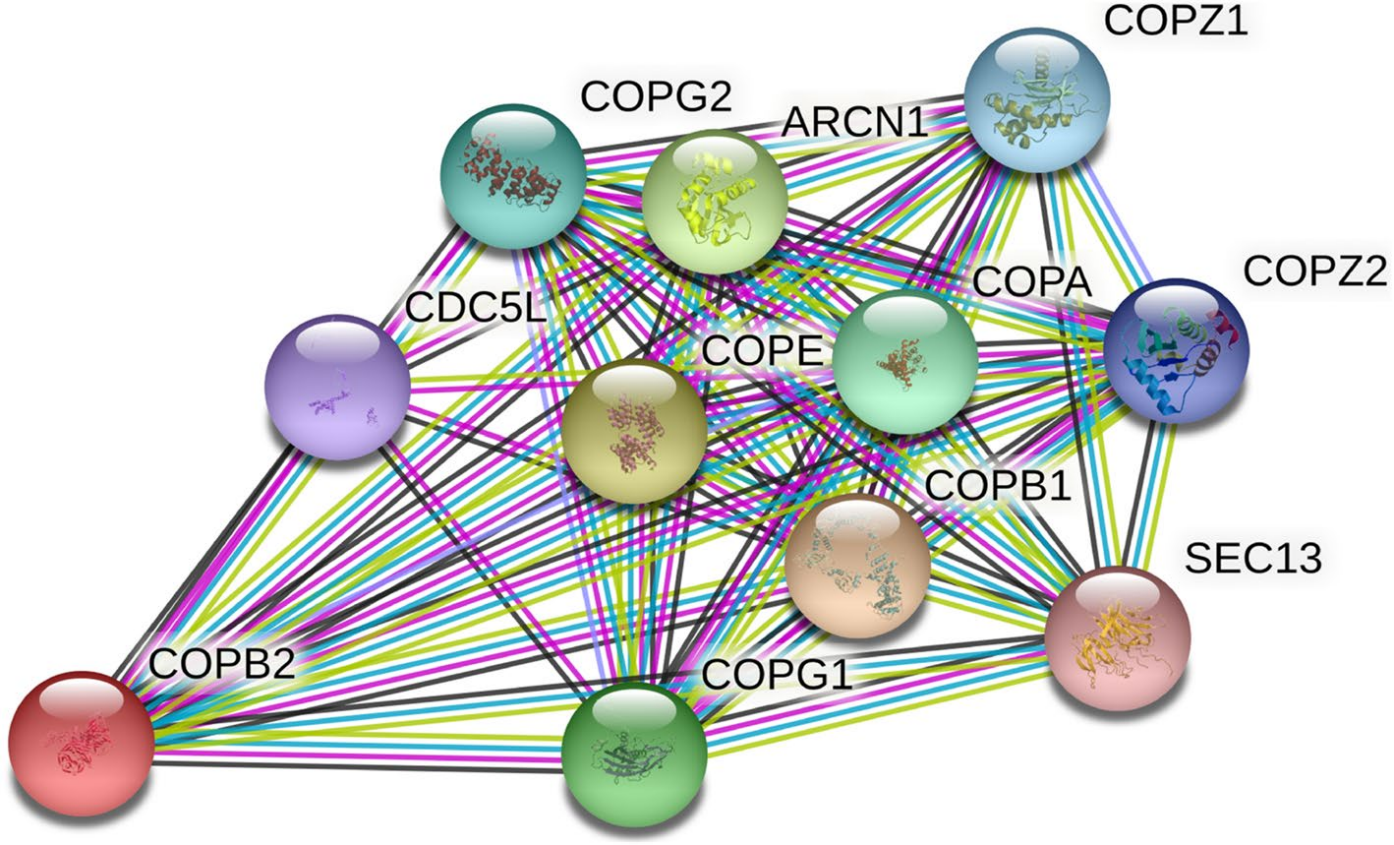
Predicted functional partners associated with COPB2 from String online website

**Fig. 3:**
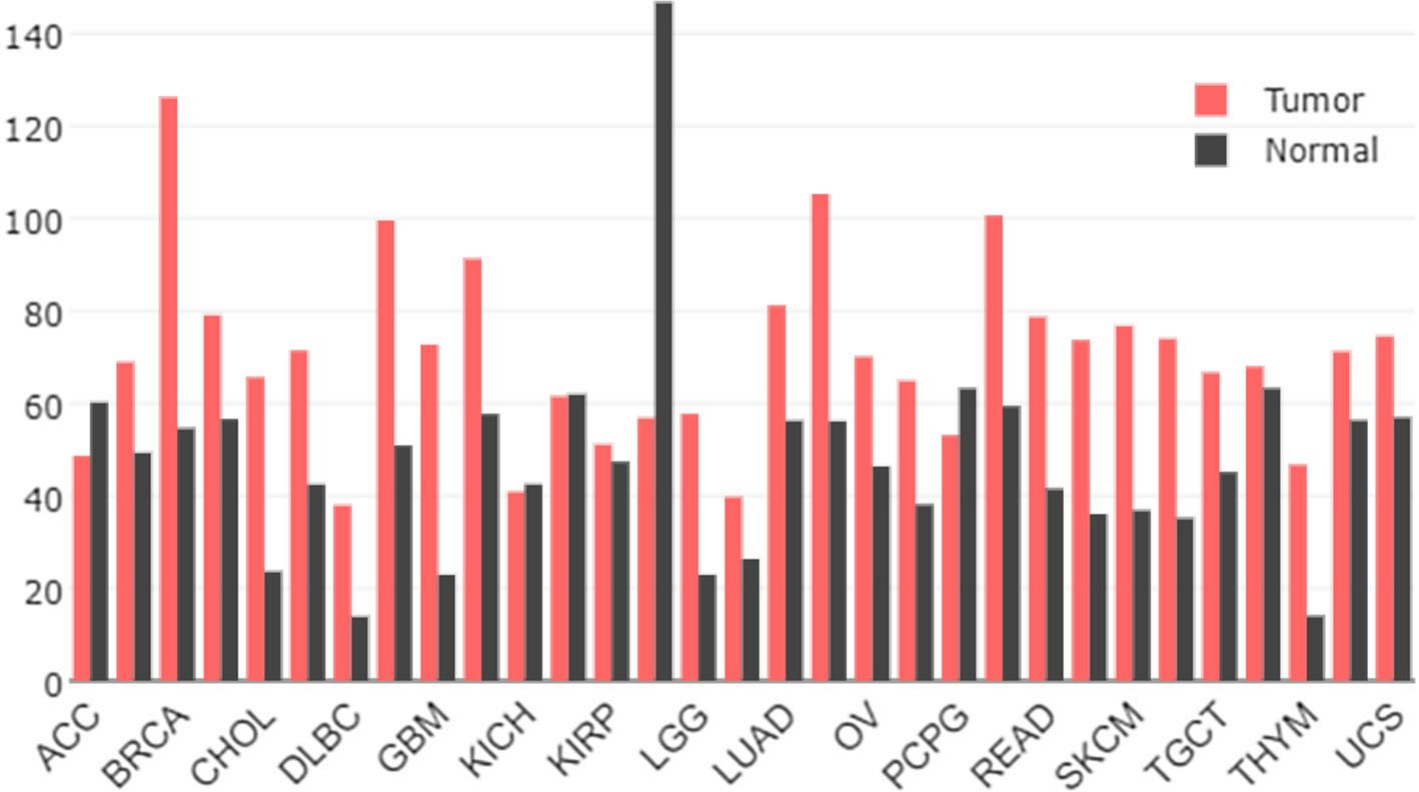
The COPB2 expression profile across all tumor samples and paired normal tissues on line database. Adrenocortical carcinoma (ACC), Breast invasive carcinoma (BRCA), Cholangio carcinoma (CHOL), Lymphoid Neoplasm Diffuse Large B-cell Lymphoma (DLBC), Glioblastoma multiforme (GBM), Kidney Chromophobe (KICH), Kidney renal papillary cell carcinoma (KIRP), Brain Lower Grade Glioma (LGG), Lung adenocarcinoma (LUAD), Ovarian serous cystadenocarcinoma (OV), Pheochromocytoma and Paraganglioma (PCPG), Rectum adenocarcinoma (READ), Skin Cutaneous Melanoma (SKCM), Testicular Germ Cell Tumors (TGCT), Thymoma (THYM), Uterine Carcinosarcoma (UCS)

The Golgi coatomer complex (MIM 601,924) constitutes the coat of non-clathrin coated vesicles and is essential for Golgi budding and vesicular trafficking. To predict the genes that interact with COPB2 and to better understand the biological role of COPB2, we used the STRING database to search for the functional partners of COPB2. The search yielded coatomer subunit beta (COPB), coatomer subunit epsilon (COPE), coatomer subunit delta (ARCN1), coatomer subunit gamma-1 (COPG1), coatomer subunit alpha (COPA), coatomer subunit gamma-2 (COPG2), coatomer subunit zeta-1 (COPZ1), coatomer subunit zeta-2 (COPZ2), cell division cycle 5-like protein (CDC5L), and protein SEC13 homolog (SEC13) (Fig. 2). Although the level of COPB2 in cancer tissues is lower than in normal tissues in adrenocortical carcinoma (ACC), kidney chromophobe (KICH), kidney renal clear cell carcinoma (KIRC), and acute myeloid leukemia (LAML), the level of COPB2 expression in most other cancer tissue types is higher than in normal tissues (Fig. 3), according to the GEPIA database.

## Future perspectives

### COPB2 and autophagy

Autophagy, which delivers cellular materials to lysosomes for degradation, leading to the basal turnover of cell components and providing energy and macromolecular precursors to cells, is another major mechanism in the progression of cancer [51]. Yamamoto et al. [52] pointed out that autophagy promoted immune evasion of pancreatic cancer by degrading the major histocompatibility complex class I (MHC-I). Furthermore, HPV16 drive cancer immune escape via NLRX1-mediated degradation of STING [53]. Thus, autophagy is an effective escape mechanism in cancer; in addition, it has already been implicated in the development of drug resistance in multiple cancer types [54, 55]. Evidence shows that autophagy caused by chemotherapeutics may boost the resistance of cancer cells to paclitaxel, tamoxifen, epirubicin, or trastuzumab [55]. However, the connection between *COPB2* and autophagy has not yet been described. Therefore, we suggest that the regulatory role of *COPB2* in autophagy should be considered in future studies.

### COPB2 and other diseases

*COPB2* is also involved in other diseases. Based on a genome-wide association study, *COPB2* is a susceptibility gene for Kawasaki disease [56], and *COPB2* homozygous mutations have been associated with microcephaly [57, 58]. *COPB2* has also been identified as a vitamin D-regulated gene, along with other new candidate vitamin D response elements that have demonstrated importance for transcriptional regulation, immune function, stress response, and DNA repair [59]. Notably, knockdown of *COPB2* is detrimental to parasitic infection, thereby inhibiting malaria [60]. Meanwhile, as one of the candidate genes for neuronal function and mu opioid receptor expression, as revealed by whole-genome expression profiling, *COPB2* is implicated in modified neuronal development, central nervous system patterning processes, differentiation and dopaminergic neurotransmission, the serotonergic signaling pathway, and glutamatergic neurotransmission [61]. In addition to its benefits to human health, targeting *COPB2* can be beneficial to certain aspects of breeding and animal husbandry. Knocking down COPB2 had been shown to destroy the integrity of the epithelial cell membrane and contribute to increased mortality of *Tetranychus urticae* [62], *Aedes aegypti* [63], *Lepeophtheirus salmonis* [64]. It has therefore been recognized as a target candidate for new pest control methods.

### COPB2 and animal models

Based on the currently available literature, we found that studies on *COPB2* were mostly limited to the cellular level. The only research we found in vivo was the one conducted by An et al. [14], which demonstrated the function of COPB2 silencing in the xenograft nude mouse model. They proposed that silencing COPB2 using the Lv‑shCOPB2 vector significantly inhibited the tumorigenicity of gastric cancer cells, and the total radiant efficiency of mice in the Lv‑shCOPB2‑infected group was markedly reduced compared with that in the Lv‑shCtrl‑infected group. To the best of our knowledge, more in vivo studies must be carried out before *COPB2* targeting can be fully applied in the clinical stage. *COPB2* has been implicated in different aspects of tumorigenesis in in vitro studies. It is therefore considered as a potential biomarker for cancer progression and cancer treatment. Hence, studies in animal models must be performed to support the use of *COPB2* in cancer therapy, diagnosis and follow up.

### COPB2 and new technologies

In recent years, researchers have devoted more energy to understanding the underlying mechanisms of cancer etiology to identify new drug targets. It has long been recognized that cancer is a heterogeneous disease, and genome changes play a crucial role in the occurrence of this disease. In the past few years, many new technologies have been used in cancer identification and treatment. For example, with the development of technologies such as single cell sequencing, microarray chips, and big data, other regulatory factors upstream of *COPB2* can also be identified. Furthermore, single cell sequencing can accurately determine the number of gene copies in a single nucleus and can therefore be an accurate test to estimate *COPB2* copy numbers to reduce false positive results and resolve issues on heterogeneity in future studies.

## Conclusion

Here, we summarize the emerging roles of coatomer protein COPB2 in cancer development and progression in light of the hallmarks of cancer. *COPB2* is viewed as a vital oncogene in many cancer types that regulates multiple biological behaviors of tumor cells, including proliferation, survival, tumorigenesis, invasion, and metastasis. However, current research on the role of *COPB2* is still lacking, and many details will be worth exploring in the future.
